# Socioeconomic Inequalities in Child Malnutrition in Bangladesh: Do They Differ by Region?

**DOI:** 10.3390/ijerph17031079

**Published:** 2020-02-08

**Authors:** Mohammad Monirul Hasan, Jalal Uddin, Mohammad Habibullah Pulok, Nabila Zaman, Mohammad Hajizadeh

**Affiliations:** 1 Food and Agriculture Organization of the United Nations (FAO), House-37, Road-8, Dhaka 1205, Bangladesh; 2 Department of Epidemiology, University of Alabama at Birmingham, USA, Birmingham, AL 35233, USA; 3 Nova Scotia Health Authority, 5955 Veteran’s Memorial Lane, Halifax, NS B3H 2E1, Canada; 4 Institute for Research, Data and Training (NB-IRDT), University of New Brunswick, 38 Dineen Drive, Fredericton, NB E3B 5A3, Canada; 5 School of Health Administration, Dalhousie University, Sir Charles Tupper Medical Building, 5850 College Street, 2nd Floor, Halifax, NS B3H 4R2, Canada

**Keywords:** stunting, underweight, socioeconomic status, inequalities, regional variation, Bangladesh

## Abstract

Socioeconomic inequality in child malnutrition is well-evident in Bangladesh. However, little is known about whether this inequality differs by regional contexts. We used pooled data from the 2011 and 2014 Bangladesh Demographic and Health Survey to examine regional differences in socioeconomic inequalities in stunting and underweight among children under five. The analysis included 14,602 children aged 0–59 months. We used logistic regression models and the Concentration index to assess and quantify wealth- and education-related inequalities in child malnutrition. We found stunting and underweight to be more concentrated among children from poorer households and born to less-educated mothers. Although the poverty level was low in the eastern regions, socioeconomic inequalities were greater in these regions compared to the western regions. The extent of socioeconomic inequality was the highest in Sylhet and Chittagong for stunting and underweight, respectively, while it was the lowest in Khulna. Regression results demonstrated the protective effects of socioeconomic status (SES) on child malnutrition. The regional differences in the effects of SES tend to diverge at the lower levels of SES, while they converge or attenuate at the highest levels. Our findings have policy implications for developing programs and interventions targeted to reduce socioeconomic inequalities in child malnutrition in subnational regions of Bangladesh.

## 1. Introduction

Child malnutrition is a significant global health problem, and it remains a major threat to the reduction of poverty and the achievement of sustainable development. Child malnutrition accounts for almost 3.1 million child deaths worldwide and is responsible for 45% of under-five child deaths [1,2]. Child malnutrition significantly affects early childhood cognitive development [3,4]. It is also responsible for school absenteeism and hinders prospects of better earnings and productivity in adulthood [5,6,7,8]. The consequences of malnutrition can also pass to the next generations as malnourished mothers give birth to infants who struggle to develop and thrive [9,10,11].

Despite the rapid improvement of social and health indicators, poor maternal and child nutrition is highly prevalent in Bangladesh. According to recent estimates, about 36% of children under five were stunted, and 33% were underweight in 2018 [12]. Half of all pregnant women and 40% of non-pregnant women suffer from anemia [12]. About one-fifth of adult women are underweight (e.g., BMI < 18.5) [12]. Further, 59% of girls get married by the age of 18 [12]. An early age at marriage and adolescent motherhood have adverse consequences for the nutritional outcomes of both mothers and children. Prior studies documented that children born to adolescent and malnourished mothers, in Bangladesh and elsewhere, have an increased likelihood of neonatal death, low birthweight, nutritional deficit, and retarded growth [13,14,15,16]. Thus, poor maternal nutrition and disadvantaged sociodemographic processes produce intergenerational cycles of malnutrition, morbidity, and mortality [17].

Studies of social determinants of health documented a strong socioeconomic gradient in child and adult health [18,19,20]. There is a well-established inverse association between parental socioeconomic status (SES) and children’s health and nutritional status, regardless of how SES is measured [9,21,22,23,24]. Parental education plays a crucial role in children’s nutritional intake as well-educated parents tend to be better caregivers [24,25,26]. Likewise, lack of parental education inevitably creates misinformation about micronutrient requirements and food intake for children, which, in turn, contributes to malnutrition [25,26,27]. Household economic affluence is an essential enabling resource for child health. In particular, household wealth offers leverage for ensuring the supply of nutritional foods and health-enhancing material resources in the household [28]. Therefore, it is likely that adverse child health outcomes, including malnutrition outcomes, tend to be clustered at the lower end of the household wealth spectrum.

Although the protective role of SES is widely acknowledged in current public health research, it is less clear how the individual-level SES interacts with macro-level factors such as community or regional contexts, especially in low-income settings. A growing body of research, especially in the context of developed countries, indicates that regional or community contexts moderate or mitigate the effects of individual-level SES on child and adult health outcomes. These studies commonly demonstrate that growing up in deprived communities (characterized by adverse social, ecological, and political contexts) exacerbates the effects of individual-level SES on child and adult health [29,30]. These studies implicate that the local socioeconomic resources and provision of community facilities such as drinking water, sanitation, transportation, accessibility to healthcare, and markets may condition the expression of individual-level SES factors that are associated with health and nutrition-related behaviours and practices [31,32].

**Figure 1:**
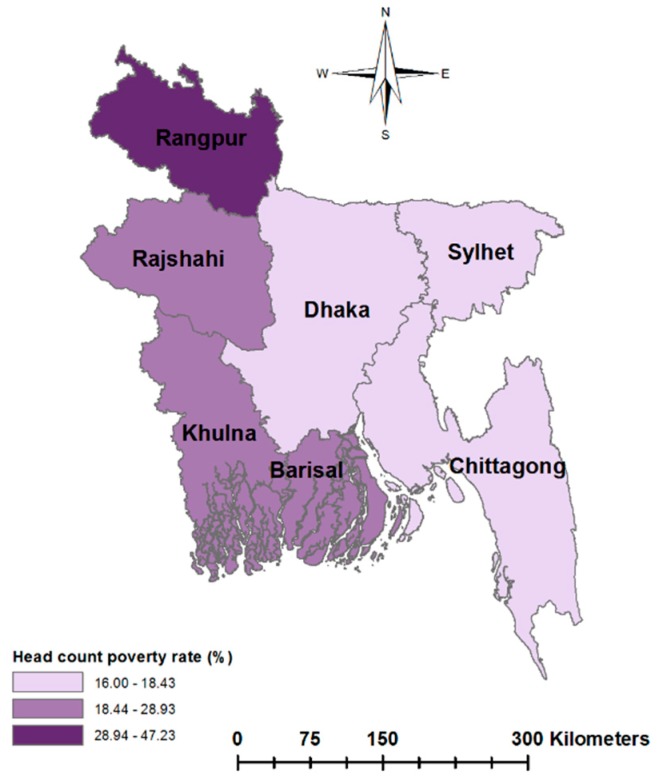
Poverty rates across the regions of Bangladesh, based on Household Income and Expenditure Survey 2016–2017.

**Figure 2:**
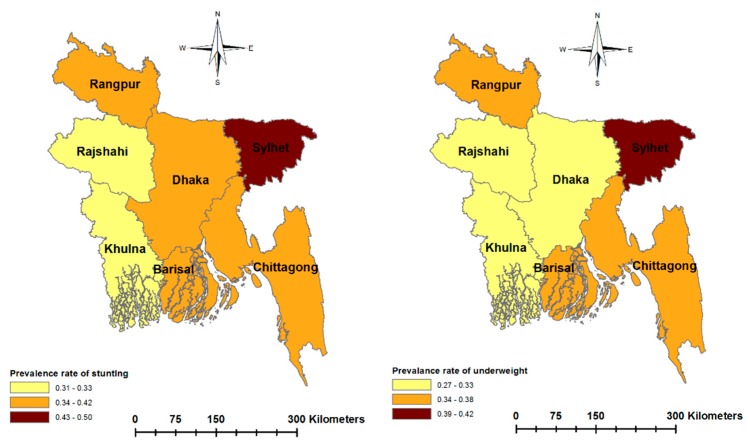
Prevalence of child malnutrition (in percentage) across the regions of Bangladesh, calculated from the 2011 and 2014 Bangladesh Demographic and Health Survey.

In the pursuit of promoting equity in health and equitable gains from development efforts in many low-income countries, policymakers strongly emphasize the reduction of regional inequality in health and wellbeing. Examining the regional variation in child malnutrition may provide a new lens to policymakers to design interventions specific to regional inequality in SES. Some studies have reported significant geographic variation in the prevalence of undernutrition in developing countries [33,34,35]. For example, despite having lower poverty rates in the eastern part of Bangladesh, the malnutrition rates are higher in this part of the country compared to the north-western region [36,37]. This contradiction is depicted in Figure 1 and Figure 2, which show the poverty rates and child malnutrition prevalence across the regions in Bangladesh. Although the eastern region has lower income poverty, this region has unique non-income characteristics. For example, the Chittagong region has many hill tracks and coastal belts and the Sylhet region has ecologically vulnerable areas characterized by physical remoteness, wetland ecosystems and social conservatism [36,38]. These regional factors may potentially constitute non-income barriers to the nutritional status of children.

To date, some studies [22,23,39] have documented socioeconomic inequalities in child malnutrition in Bangladesh. However, no study has examined the extent to which the regional contexts in Bangladesh may condition the SES gradient in child malnutrition. To fill this gap in the literature, we investigated regional variations in socioeconomic inequalities in the prevalence of stunting and underweight among children under five in Bangladesh.

## 2. Materials and Methods

### 2.1. Data and Samples

We pooled data from two recent rounds of the Bangladesh Demographic and Health Survey (BDHS) conducted in 2011 and 2014. The BDHS is a nationally representative survey on maternal and child health, mortality, fertility, family planning, and nutrition. It also has an extensive collection of sociodemographic and socioeconomic variables such as education, household assets, and occupation. The BDHS used a multistage cluster sampling technique, which allows estimations at both national and regional levels. A detailed description of the survey methodology, sampling design, and primary finding of the BDHS is available in the National Institute of Population Research and Training (NIPORT) report [37]. We used data from the child module of all children aged 0 to 59 months.

The BDHS collected data from ever-married women aged 15 to 49 years and their children aged 0–59 months using pre-tested structured questionnaires. The response rate in the BDHS survey was about 98% [37]. Trained interviewers used lightweight Seca digital scales and standard wooden boards to measure the weight and height of the mothers and their children [17,37]. Recumbent length was measured for children aged 0–24 months and standing height was recorded for all other children. The analysis excluded about 18% of the children who had incomplete or missing information on height, weight, and age. The final analytical sample included 14,602 children with complete information on anthropometric data and all other variables used in the analysis.

### 2.2. Measures of Child Malnutrition

Child malnutrition is the outcome variable of this study. In the BDHS, child malnutrition is measured by standard anthropometric indicators of height-for-age (HAZ) z-scores and weight-for-age (WAZ) z-scores as defined by the World Health Organization (WHO) [40]. Stunting (HAZ) and underweight (WAZ) are the two anthropometric measures that are commonly used as a proxy for child malnutrition [41]. However, there is a difference between these two measures. Stunting is an indicator of low height by age while underweight measures low weight by age. Stunting is the long-term measure of child malnutrition. A child is stunted or underweight if the child’s HAZ (or WAZ) z-score falls two standard deviations (SD) below the median of the WHO reference population [22,40]. The study used a binary indicator of stunting (whether a child is stunted or not) and underweight (whether a child underweight or not).

### 2.3. Explanatory Variables

We followed previous studies to model the relationship between child malnutrition and its associated factors [42,43]. The explanatory variables included in this study are wealth index, region of residence, place of residence, mother’s education, father’s education, mother’s age, child characteristics, (age, sex, twin child, and birth order) and the number of living children in the household. Parental education was a continuous variable of completed years of schooling.

The BDHS does not collect data on income. Instead, the BDHS collects a wide range of household assets and dwelling characteristics, ranging from ownership of a radio/TV, a refrigerator, farmland, farming animals, materials used for the construction of houses, water, and sanitation infrastructure and so on [44,45]. The BDHS constructs a wealth index variable based on the amount and type of such assets and household characteristics by applying a principal component analysis (PCA). Broadly, PCA produces a range of scores for each asset and dwelling characteristics and gives each household a summative wealth score based on its ownership of amount and type of assets. The underlying assumption of the wealth index is that there is a continuum of the economic status of the households in a given country and a particular household’s cumulative wealth represents its relative position in such a continuum of the economic status. Finally, the cumulative wealth scores are divided into five wealth quintiles: the lowest 20% represents the lowest wealth quintile group and the highest 20% represents the highest wealth quintile group. Households were divided into five equal groups to produce sufficient samples and to ease the interpretation of the results. The details of the methodology and statistical approach used for constructing the wealth index are described elsewhere [44,45].

### 2.4. Data Analysis

We used multivariable logistic regression models to examine the associations of wealth index and education with two outcome variables (stunting and underweight). We included interaction terms between wealth, education, and region in the adjusted models to assess whether associations of wealth and education with malnutrition outcomes differed by region. We employed the Concentration index (CI) as the summary measure of socioeconomic inequality to quantify the degree of wealth- and education-related inequalities in stunting [46]. The value of the CI ranges from −1 to +1 [41]. When the CI is negative (positive), it indicates a disproportionate concentration of stunting/underweight among the children from households with low (high) wealth and less (more) educated mothers [43]. A zero value of the CI shows no socioeconomic inequality in child malnutrition.

We applied Wagstaff’s normalization (Wagstaff Index or WI) and Erreygers’s correction (Erreygers Index or EI) to take into account the binary nature of outcome variables in this study [47,48]. The analysis of regional variation in inequality was conducted using the Erreygers’s correction as it considers a change in the mean of the outcome variables across the studied regions [46]. All the estimates were adjusted using the survey design of the BDHS, which includes sampling weight, strata, and clusters. All the statistical analyses were performed using Stata software (version 15.1, StataCorp, College Station, Tex).

### 2.5. Ethical Approval

The Bangladesh Demographic and Health Survey received ethical approval from the Inner City Fund (ICF) Macro Institutional Review Board, Maryland, USA and the National Research Ethics Committee of Bangladesh Medical Research Council (BMRC), Dhaka, Bangladesh. Each participant gave informed consent before participating in the survey. The de-identified data for this study were obtained from the DHS online [49]. Institutional ethical approval was not necessary as the study was conducted on anonymous public-use data, which had no identifiable information on the survey respondents.

## 3. Results

Table 1 presents the prevalence of stunting and underweight across the regions by household wealth quintiles and mother’s education. Overall, the proportion of stunted and underweight children was the highest in Sylhet (50% and 42%), followed by Barisal (42% and 38%). Rates of child malnutrition were the lowest in Khulna. In all regions, children from the poorest households had a higher prevalence of stunting and underweight. For example, the prevalence of stunting was about 63% in the poorest wealth quintile households, while it was about 26% in the highest wealth quintile households in Sylhet. There was also a similar variation in the prevalence of stunting and underweight among children by mother’s education. For example, the prevalence of underweight was 51% among the children of mothers with no education in Barisal. On the other hand, the prevalence of underweight was about 35% among the children of mothers with no education in Rangpur. The prevalence of stunting was about 15% among the children of mothers with higher education, while it was about 60% among children of mothers with no education in Sylhet.

The multivariable logistic regression analysis shows a significant association between child undernutrition and wealth index (Table 2). Children from households in higher wealth quintiles had a lower likelihood of being stunted and underweight. For example, the odds of being stunted was 60% lower (odds ratio (OR): 0.40, 95% confidence interval (CI): 0.33–0.49) among the children from the wealthiest quintile compared to the children from the poorest wealth quintile. Malnutrition was also significantly associated with region. For example, compared to Khulna, the odds of stunting were about 1.4 (OR: 1.44, 95% CI: 1.20–1.73) times as likely in Barisal. The likelihood of children underweight in Sylhet was about 60% higher (OR: 1.59, 95% CI: 1.31–1.94) compared to Khulna. Parental education had a negative relationship with the stunting and underweight status of children. The likelihood of stunting and underweight decreased by about 15% and 9%, respectively, in 2014 compared to 2011.

Table 3 reports the estimated wealth- and education-related inequalities in child malnutrition in Bangladesh. Negative and statistically significant values of all the indices indicate that stunting and underweight were more concentrated among children from poorer households and less-educated mothers.

**Figure 3:**
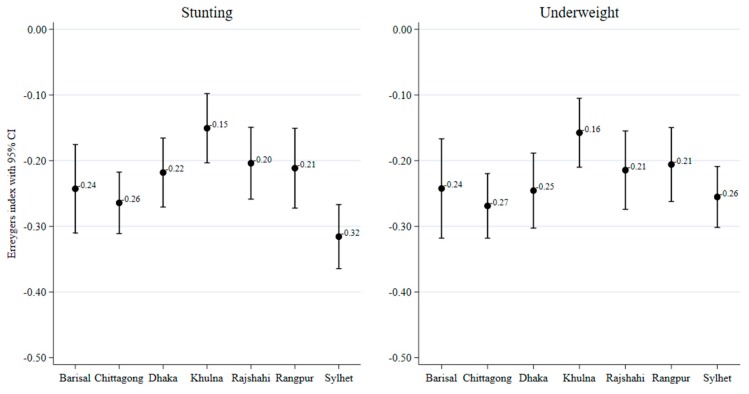
Regional variation in wealth-related inequalities in malnutrition, measured by the Erregerys Index.

**Figure 4:**
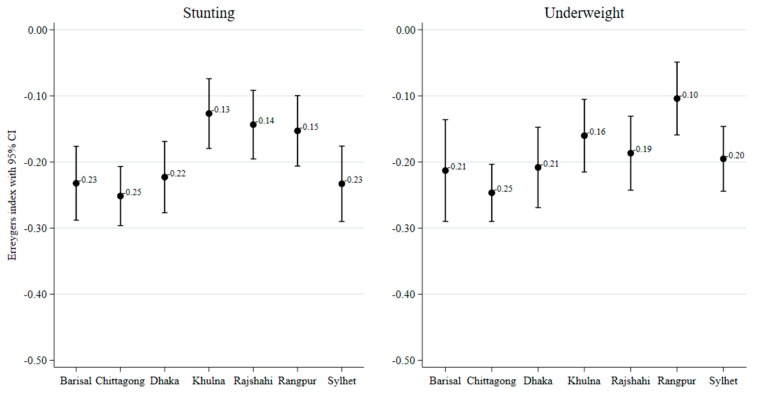
Regional variation in education-related inequalities in malnutrition, measured by the Erregerys Index.

**Figure 5:**
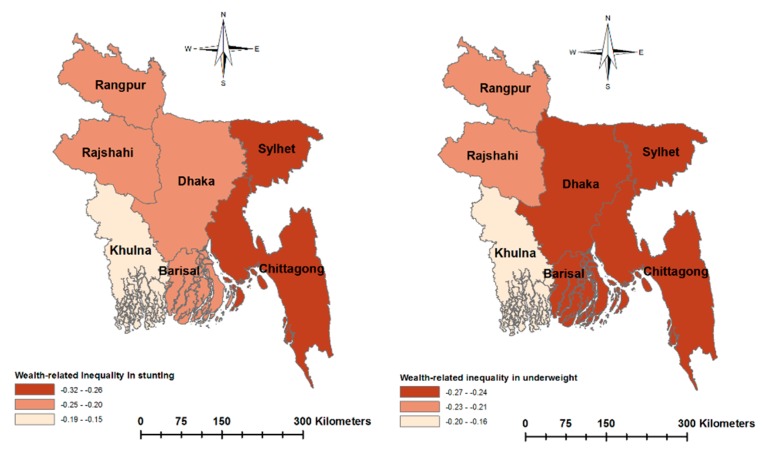
Wealth-related inequalities in child malnutrition across the regions of Bangladesh.

**Figure 6:**
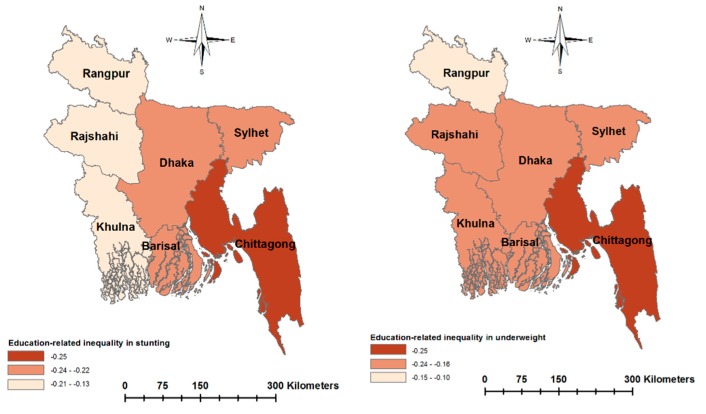
Education-related inequalities in child malnutrition across the regions of Bangladesh.

Figure 3 and Figure 4 present socioeconomic inequalities in child malnutrition (estimated by the EI) across the seven regions of Bangladesh. The negative values of EIs across all regions indicate a higher concentration of malnutrition among children of poor households and with less-educated mothers. The extent of inequality varied across the regions. Wealth-related inequality in stunting was the highest in Sylhet and the lowest in Khulna. Wealth-related inequality in underweight was the lowest in Khulna. Education-related inequality in stunting was the highest in Chittagong and the lowest in Khulna. Similarly, education-related inequality in underweight was the highest in Chittagong and the lowest in Rangpur. Figure 5 and Figure 6 show the east–west divide in wealth- and education-related child malnutrition in Bangladesh.

**Figure 7:**
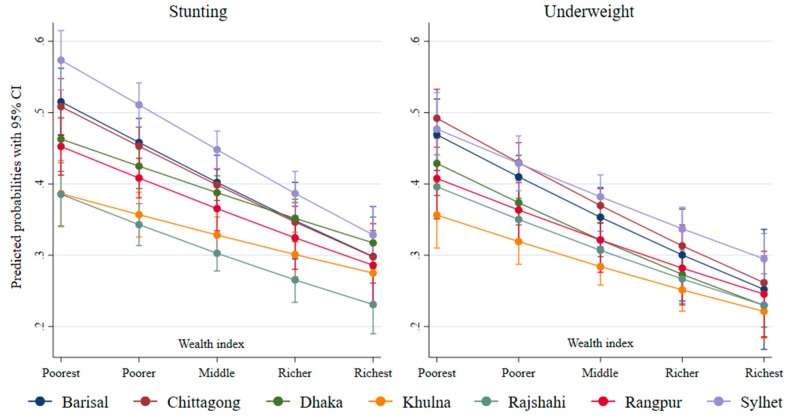
Predicated probabilities from the interactions between wealth and region.

**Figure 8:**
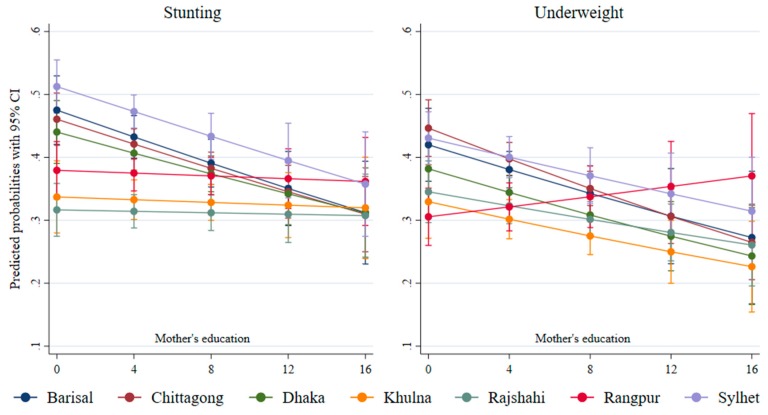
Predicated probabilities from the interactions between mother’s education and region.

Figure 7 and Figure 8 depict the predicted probabilities of stunting and underweight by wealth and education across the regions. Non-parallel predicted probability suggests that the probability of stunting by household wealth was associated with region (Figure 7). It shows that the probability of being a stunted child was the highest in the poorest quintile and lowest in the wealthiest quintile. The predicted probability of being stunted was the highest in Sylhet and the lowest in Rajshahi among the children from the poorest households. However, this difference in the predicted probabilities almost disappeared in the wealthiest quintile in all regions. The flatter predicted probability curve for Khulna indicates lower wealth-related inequality in stunting and underweight in this region compared to other regions.

The approximately flat predicted probability curves for the three western regions, such as Rangpur, Rajshahi, and Khulna, show that the level of inequality was not responsive to the level of mother’s education (Figure 8). However, the other eastern regions, such as Chittagong and Sylhet, the southern region Barisal, and the central region Dhaka showed a negative association between education and child nutrition status. Education-related inequality in stunting was the highest in the Sylhet region. There was an inverse relationship between underweight and mother’s education across the regions, except for Rangpur (Figure 8). The downward predicted probabilities indicate a negative association of education with underweight. Sylhet had the highest level of inequality in underweight than Khulna and Rajshahi.

## 4. Discussion

This study examined socioeconomic inequalities in child malnutrition and whether these inequalities differ by subnational regions in Bangladesh. Results suggested that stunting and underweight were more concentrated among children from poorer households and those born to less-educated mothers in Bangladesh. We found significant wealth- and education-related inequalities in child malnutrition in the north-eastern (e.g., Sylhet) and southern and eastern regions (e.g., Barisal and Chittagong) compared to other regions. Further, there was a differential protective effect of mother’s education and household wealth across the regions in Bangladesh. The regional differences in the effects of SES tend to diverge at the lower levels of SES and converge or attenuate at the highest levels.

Our analysis found some notable trends in the prevalence of stunting and underweight and how they differ by the subnational regions. The prevalence of stunting and underweight was disproportionately higher in southern and eastern regions, including Barisal, Chittagong, and Sylhet. Earlier studies in Bangladesh also found similar regional patterns in the prevalence of stunting and underweight. In particular, prior studies noted that eastern and south-eastern regions in Bangladesh have the highest concentration of severe child wasting and stunting, with the severity of wasting exceeding the WHO critical threshold level [34].

Regarding the socioeconomic inequalities in child nutritional outcomes, we found a higher concentration of stunting and underweight outcomes among low SES children and these inequalities varied across the regions. The highest extent of wealth-based inequality in stunting was found in Sylhet for stunting and it was the higest in Chittagong for underweight. In contrast, the lowest levels of wealth-related inequalities in stunting and underweight were found in Khulna. Education-related inequalities in stunting and underweight also varied across the regions. The highest inequality in stunting was observed in Sylhet and the lowest was observed in Rajshahi. Education-related inequality in underweight was the highest in Chittagong and the lowest in Rangpur.

Parental education and household wealth (the two measures of SES) appeared to be the most robust determinants of child malnutrition outcomes in the regression analysis. The findings imply that children born to parents with higher levels of education and wealth were the most advantaged in terms of having favourable nutritional status. Although parental SES measures are protective factors for child health, in Bangladesh and other developing countries [16,22,50,51], we observed considerable heterogeneity in the effects of these protective factors across the regions in Bangladesh. For instance, the predictive probabilities, computed from the adjusted models, broadly demonstrated that both household wealth and mother’s education are negatively associated with outcomes across the regions. However, regional differences in the predicted estimates of the associations of SES indicators with outcomes tend to be larger at the lowest levels of SES indicators and converge or attenuate at the highest levels. These findings imply that most advantaged sections of the population had favourable child health outcomes irrespective of their region of residence.

The observed regional differences in our analysis fairly corroborate with a few recent studies in Bangladesh and elsewhere. Studies in Bangladesh have shown that wealth-based inequalities in health, especially in maternal health and child mortality, were relatively larger in the eastern and south-eastern regions in comparison to the western regions in Bangladesh [38,52]. These studies identified the spatial clustering of health disadvantages. They concluded that higher inequalities in the southern and south-eastern regions in Bangladesh may reflect the region-specific unique characteristics, including physical remoteness, lack of transportation, poor road infrastructure, wetland ecosystem, and ethnic conflicts, which have implications for access to food and health-enhancing resources [36,53,54]. Similarly, an analysis of five African countries has shown that although there is a strong individual-level SES gradient in child malnutrition, community SES sometimes has an independent effect [29]. Further, community context or regional inequality in socioeconomic resources can modify the effects of individual and household SES such that community or regional context appears to be the distal precondition for the proximal individual-level SES to render its protective effects on child health [29,55,56].

This study has some limitations. First, our findings do not allow us to infer any causal relationship between SES and child malnutrition outcomes due to the cross-sectional study design. Second, we could not control for other regional factors that could potentially shape the associations between SES and outcomes in our analysis. Future research may investigate the association of other structural and contextual factors, such as community-level poverty, physical and financial barriers to health facilities, and an array of cultural and local contexts contributing to the regional differences in child health.

## 5. Conclusions

Child malnutrition is a major public health issue in Bangladesh. Reducing socioeconomic inequalities in child health has remained to be a critical challenge for policymakers. Subnational regional variation in socioeconomic inequalities in child malnutrition in Bangladesh requires further public health attention. Understanding regional differences in socioeconomic inequalities could provide useful information to help formulate strategies to reduce social inequalities in malnutrition.

## Figures and Tables

**Table 1:** Prevalence of child malnutrition by wealth and education across the regions in Bangladesh.

		Barisal	Chittagong	Dhaka	Khulna	Rajshahi	Rangpur	Sylhet
Stunting	Overall	0.42	0.40	0.39	0.31	0.33	0.40	0.50
	Wealth quintiles							
	Poorest	0.59	0.58	0.49	0.42	0.42	0.49	0.63
	Poorer	0.40	0.48	0.46	0.32	0.40	0.43	0.56
	Middle	0.41	0.39	0.41	0.32	0.31	0.37	0.49
	Wealthier	0.33	0.32	0.39	0.31	0.21	0.29	0.39
	Wealthiest	0.19	0.25	0.23	0.19	0.17	0.17	0.26
	Mother’s education							
	No education	0.57	0.52	0.49	0.45	0.40	0.42	0.60
	Primary	0.48	0.50	0.45	0.34	0.37	0.47	0.54
	Secondary	0.37	0.32	0.33	0.30	0.30	0.38	0.40
	Higher	0.29	0.24	0.20	0.20	0.16	0.20	0.15
Underweight	Overall	0.38	0.37	0.32	0.27	0.33	0.36	0.42
	Wealth quintiles							
	Poorest	0.53	0.55	0.47	0.35	0.42	0.46	0.53
	Poorer	0.39	0.45	0.39	0.34	0.40	0.37	0.46
	Middle	0.34	0.37	0.32	0.29	0.34	0.29	0.43
	Wealthier	0.23	0.28	0.30	0.25	0.22	0.24	0.31
	Wealthiest	0.25	0.22	0.17	0.14	0.16	0.21	0.26
	Mother’s education							
	No education	0.51	0.52	0.44	0.43	0.43	0.35	0.51
	Primary	0.45	0.45	0.37	0.33	0.41	0.43	0.45
	Secondary	0.33	0.29	0.26	0.25	0.27	0.35	0.35
	Higher	0.23	0.21	0.15	0.17	0.19	0.20	0.13

**Table 2:** Multivariate logistic regression results on the correlates of child malnutrition.

	Stunting		Underweight	
	Adjusted Odds Ratio	95% Confidence Interval	Adjusted Odds Ratio	95% Confidence Interval
Region (Ref: Khulna)				
Barisal	1.44 ***	(1.20–1.73)	1.43 ***	(1.18–1.72)
Chittagong	1.39 ***	(1.19–1.61)	1.52 ***	(1.29–1.80)
Dhaka	1.34 ***	(1.14–1.56)	1.23 **	(1.03–1.45)
Rajshahi	0.89	(0.75–1.06)	1.12	(0.93–1.34)
Rangpur	1.20 **	(1.02–1.42)	1.19	(0.93–1.51)
Sylhet	1.80 ***	(1.52–2.11)	1.59 ***	(1.31–1.94)
Wealth quintiles (Ref: Poorest)				
Poorer	0.85 **	(0.75–0.97)	0.81 ***	(0.71–0.93)
Middle	0.74 ***	(0.65–0.85)	0.67 ***	(0.59–0.77)
Wealthier	0.63 ***	(0.54–0.73)	0.53 ***	(0.45–0.61)
Wealthiest	0.40 ***	(0.33–0.49)	0.38 ***	(0.32–0.45)
Mother’s education (years)	0.97 ***	(0.95–0.99)	0.97 ***	(0.95–0.99)
Father’s education (years)	0.96 ***	(0.95–0.98)	0.97 ***	(0.96–0.99)
Mother’s age	0.97 ***	(0.95–0.98)	0.98 ***	(0.97–0.99)
Rural (Ref: Urban)	0.83 ***	(0.73–0.95)	0.98	(0.87–1.10)
Child’s gender (Ref: Male)	0.99	(0.91–1.08)	1.12 **	(1.02–1.22)
Age of child (months)	1.02 ***	(1.02–1.02)	1.02 ***	(1.02–1.02)
Twin child (Ref: No)	1.84 ***	(1.21–2.80)	1.96 ***	(1.27–3.02)
Birth order	1.09 **	(1.01–1.17)	1.09 **	(1.01–1.18)
Number of children	1.04	(0.96–1.13)	0.97	(0.88–1.06)
Year—2014 (Ref: 2011)	0.85 ***	(0.77–0.93)	0.91 *	(0.81–1.01)
Observations	14,602			

Significance level: *** *p* < 0.01, ** *p* < 0.05, * *p* < 0.1.

**Table 3:** Wealth- and education-related inequalities in child malnutrition in Bangladesh.

Stunting		95% Confidence Interval	Underweight	95% Confidence Interval
Wealth-related inequality				
CI	−0.147 ***	(−0.163, −0.131)	−0.172 ***	(−0.191, −0.154)
WI	−0.240 ***	(−0.266, −0.215)	−0.263 ***	(−0.292, −0.235)
EI	−0.228 ***	(−0.253, −0.204)	−0.238 ***	(−0.264, −0.212)
Education-related inequality				
CI	−0.139 ***	(−0.154, −0.124)	−0.148 ***	(−0.166,−0.129)
WI	−0.227 ***	(−0.251, −0.202)	−0.225 ***	(−0.254, −0.197)
EI	−0.215 ***	(−0.239, −0.192)	−0.204 ***	(−0.229, −0.178)

Level of significance * *p* < 0.10, ** *p* < 0.05, *** *p* < 0.00. CI = Concentration index, WI = Wagstaff index, EI = Erreygers Index.
